# Responses of Symbiodiniaceae Shuffling and Microbial Community Assembly in Thermally Stressed *Acropora hyacinthus*

**DOI:** 10.3389/fmicb.2022.832081

**Published:** 2022-04-01

**Authors:** Wentao Zhu, Xiangbo Liu, Ming Zhu, Xinke Li, Hongyang Yin, Jianzhong Huang, Aimin Wang, Xiubao Li

**Affiliations:** ^1^College of Ecology and Environment, Hainan University, Haikou, China; ^2^State Key Laboratory of Marine Resource Utilization in South China Sea, Hainan University, Haikou, China; ^3^College of Marine Science, Hainan University, Haikou, China

**Keywords:** scleractinian coral, coral holobiont, elevated temperatures, Symbiodiniaceae shuffling, stochastic processes, bacterial species coexistence

## Abstract

Although the importance of coral holobionts is widely accepted, the relationship between the flexibility of the microbial structure and the coral host is very complicated. Particularly, the community dynamics of holobionts and the stability of host–microbe interactions under different thermal stresses remain largely unknown. In the present study, we holistically explored the physiology and growth of *Acropora hyacinthus* in response to increased temperatures (from 26 to 33°C). We observed that bleaching corals with loss of algal symbionts reduced lipids and proteins to maintain their survival, leading to decreased tissue biomass and retarded growth. The diversity of Symbiodiniaceae and symbiont shuffling in the community structure was mainly caused by alterations in the relative abundance of the thermally sensitive but dominant clade C symbionts and low abundance of “background types.” Bacterial diversity showed a decreasing trend with increasing temperature, whereas no significant shifts were observed in the bacterial community structure. This finding might be attributed to the local adjustment of specific microbial community members that did not affect the overall metabolic state of the coral holobiont, and there was no increase in the proportion of sequences identified as typically pathogenic or opportunistic taxa. The Sloan neutral community model showed that neutral processes could explain 42.37–58.43% of bacterial community variation. The Stegen null model analysis indicates that the stochastic processes explain a significantly higher proportion of community assembly than deterministic processes when the temperature was elevated. The weak effect of temperature on the bacterial community structure and assembly might be related to an increase in stochastic dominance. The interaction of bacterial communities exhibits a fluctuating and simplistic trend with increasing temperature. Moreover, temperature increases were sufficient to establish the high stability of bacterial networks, and a non-linear response was found between the complexity and stability of the networks. Our findings collectively provide new insights into successive changes in the scleractinian coral host and holobionts in response to elevated seawater temperatures, especially the contribution of the community assembly process and species coexistence patterns to the maintenance of the coral-associated bacterial community.

## Introduction

It is widely recognized that scleractinian corals rely on their symbiotic relationship with Symbiodiniaceae (Conti-Jerpe et al., 2020), and diverse microbiota (including bacteria, archaea, fungi, viruses, and protists) and their coral hosts constitute the holobiont (Kelly et al., 2014). It is accepted that Symbiodiniaceae provide nutrition and energy to coral hosts (Thompson et al., 2014), whereas other symbiotic microorganisms play an essential role in host fitness and their ability to adapt to environmental perturbation (Peixoto et al., 2017). Changes and stress in environmental conditions can alter the microbial composition as well as the health of the holobiont (van Oppen and Blackall, 2019), and the increase in sea surface temperature (SST) dramatically contributes to the collapse of the symbiotic relationship between the coral host and Symbiodiniaceae symbionts (Glynn, 1993). After the loss of Symbiodiniaceae in coral tissues and/or the reduction of photosynthetic pigment concentration, if a stable symbiotic relationship is not reestablished, coral bleaching leads to host starvation and eventual death (Buerger et al., 2020). Large-scale coral bleaching events caused by ocean warming have significantly reduced coral coverage worldwide (Barshis et al., 2013). As a result, the structure of coral reefs and ecosystem functions worldwide are experiencing unprecedented decline and loss (Hughes et al., 2018). The death of corals caused by bleaching after ocean warming events is considered the most urgent climate-related threat to coral communities (Hughes et al., 2017). The decline in coral abundance and the overall loss of coral reef habitat have become one of the most pressing environmental problems of this era (Shinzato et al., 2011). However, coral–microbiome interactions are not yet fully understood due to the complex symbiotic relationship (Shinzato et al., 2011). Understanding how each member of the holobiont contributes to the resilience in response to elevated temperature is paramount under global warming conditions (Thompson et al., 2014). Therefore, it is necessary to explore the complex relationship and the essential role of coral-associated microbiota under healthy conditions or when homeostasis breaks down.

Understanding the mechanisms underlying microbial community assembly has recently become a key topic in ecology (Nemergut et al., 2013; Dini-Andreote et al., 2015). The assembly of species into communities determining the existence and abundance of species includes deterministic processes (such as local environmental conditions, species traits, and interspecies interactions) and stochastic processes (random birth, death, and dispersal events) (Stegen et al., 2012; Zhou and Ning, 2017). Recent studies report that stochastic processes shape microeukaryotic community assembly in a subtropical river across wet and dry seasons (Stegen et al., 2012; Zhou and Ning, 2017), and deterministic selection (the influence of mean annual temperature) dominates microbial community assembly in termite mounds (Qlca et al., 2020). Low shifts in salinity can drive deterministic assembly processes and network stability to affect the assembly of microeukaryotic plankton communities in a subtropical urban reservoir (Mo et al., 2021). The variation in soil organic matter can change the relative influence of different assembly processes on the formation of soil bacterial communities (Dini-Andreote et al., 2015). In addition, studies find that, as hosts develop from larvae to adults, non-neutral processes, such as microbe–microbe interactions, active dispersal, or selection by the host, are increased as hosts mature (Burns et al., 2016). After a long-standing debate, it is generally accepted that deterministic and stochastic processes are not mutually exclusive, and both act simultaneously to regulate the assembly of ecological communities (Jonathan et al., 2011; Tripathi et al., 2018). In contrast, our knowledge is still limited regarding the primary forces (such as stochastic or deterministic) that dominate the microbial diversity and community composition within scleractinian corals (Price et al., 2021), especially under the influence of different thermal stresses, which is essential to predict the role of coral bacterial communities in contributing to the holobiont function.

*Acropora hyacinthus* is a thermally sensitive and widespread species with a high prevalence on Pacific reefs (Ziegler et al., 2017). In addition, it has the advantages of fast growth and strong adaptability and is an excellent candidate species for coral reef restoration near Hainan Island (Xiao et al., 2018). In the current study, we aimed to employ a holistic approach to examine successive changes in the coral holobiont in response to increasing temperatures under controlled laboratory conditions. Therefore, the specific objectives of this study were (1) to determine the characteristics of the growth and physiological changes of *A. hyacinthus* under the influence of elevated SST, (2) to clarify the response of crucial coral holobiont (Symbiodiniaceae and bacteria), (3) to quantify the relative importance of deterministic and stochastic processes in coral bacterial assembly under different thermal stresses, and (4) to explore the role of microbial interactions in community assembly. Moreover, we analyzed the growth responses of the coral host *A. hyacinthus* and changes in Symbiodiniaceae symbionts, the bacterial community assembly mechanism, and the coexistence pattern under the influence of increased water temperatures. These results provide valuable insights into the mechanisms underlying the interactions between coral hosts and holobionts in response to elevated temperatures, which is very important to evaluate the adaptive ability of scleractinian coral to global warming.

## Materials and Methods

### Study Design

Divers collected 10 healthy *A. hyacinthus* colonies through scuba diving on April 24, 2021, at a depth of 3–4 m from the coral nursery in Wuzhizhou (109°45′E, 18°18′N) in Sanya, China. The SST in the sampling area was about 27°C, the pH value was between 8.03 and 8.22, and the average salinity was about 34‰. The coral samples were maintained in the seawater *in situ* and transported to the laboratory immediately. After each coral was properly chiseled into multiple experimental nubbins of suitable size, the coral nubbins were placed vertically on an acrylic plate and directly transferred to three aquaculture tanks of 680 mm × 450 mm × 360 mm (length × width × height). Coral was then allowed to acclimate for 2 weeks in a circulating indoor aquarium with approximately 270 L of synthetic seawater (26°C). The synthetic seawater was prepared by mixing artificial sea salt and pure water. The water flow was provided by a circulating pump and a wave pump in each aquarium. The aquarium lighting (6 T5HO) was maintained throughout the experiment similar to natural light (12:12 h light/dark cycle). Stable water environmental conditions were maintained: salinity 33‰∼35‰, pH 8.3∼8.4, and dissolved oxygen (DO) 7.5∼8.5. Moreover, 20% of the aquarium water was replaced every 5 days to maintain sufficient concentrations of trace elements, and the coral were not subjected to additional feeding treatments.

**FIGURE 1:**
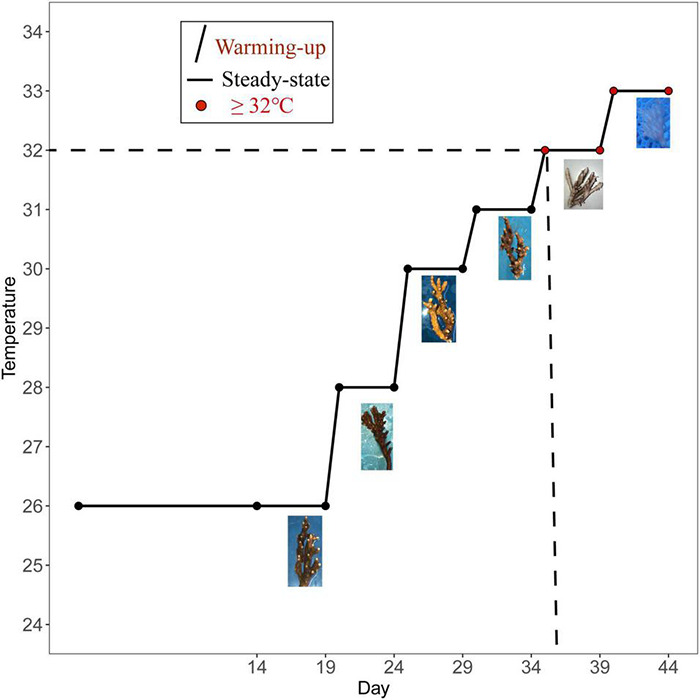
Different temperature treatments and sampling time points.

The seawater temperatures of the three tanks were gradually increased from 26 to 33°C to test the effect of gradual heat stress on coral (the temperature was elevated within a day). The coral were acclimatized at 26°C for 14 days before the heat stress and then kept at 26°C until the 19th day to collect samples. Then, the temperature was slowly increased to 28°C for 4 days. On the 24th day, it was slowly increased to 30°C and maintained for 4 days. On the 29th day, the temperature was slowly increased to 31°C and maintained for 4 days. On the 34th day, it was slowly increased to 32°C and maintained for 4 days. On the 39th day, the temperature was slowly elevated to 33°C and maintained for 4 days. The water temperature was regulated using a 500-W submersible aquarium heater, which was connected to a digital thermostat, and the temperature was slowly increased to the experimental temperature for heat stress throughout the day. The total experimental period was 44 days, and the sampling time points are shown in Figure 1. Corals from temperature treatment lasting 4 days (26, 28, 30, 31, 32, and 33°C) were collected on the corresponding fifth day, including six temperature groups of 26°C (T26 group), 28°C (T28 group), 30°C (T30 group), 31°C (T31 group), 32°C (T32 group), and 33°C (T33 group). To ensure data reliability, two samples were randomly collected from each of the three experimental pools, and six biological replicates were used to analyze physiological indexes, symbiotic family community, and bacterial community. Among them, growth rate and photosynthetic physiological indicators were measured repeatedly in 6–9 nubbins in each temperature group, and only samples from T26/T32/T33 were used for energy substance analysis and Symbiodiniaceae sequencing. Each nubbin was cut into fragments of the size required for the experiment and then immediately placed in centrifuge tubes and stored at −80°C until subsequent analysis.

### Physiological Analyses

To determine the growth rate of coral at different temperatures, the buoyancy weighing-bar method (Davies, 1989) was used to weigh randomly selected and labeled pieces of coral every 5 days, and the specific growth rates (μ) were calculated as previously described (Wijgerde et al., 2020). During the whole experiment, the chlorophyll-modulated fluorometer MINI-PAM-II (Walz, Germany) was used to non-invasively measure the maximum quantum yield (Fv/Fm) of photosystem II (PSII) within the symbiotic algae.

All physiological analyses were performed as previously described (Zhu et al., 2021). Briefly, the coral tissue was rinsed with a Waterpik dental scaler (Waterpik) containing filtered seawater (0.45 μm, Whatman, United Kingdom), and the zooxanthellae density was determined using a hemocytometer under a microscope. The coral sample was dried to constant weight at 60°C for at least 24 h and then burned in a muffle furnace at 500°C for 4 h, and the tissue biomass was then determined (Fitt et al., 2000). According to the manufacturer’s instructions, the total protein content of coral homogenate was quantitatively determined using a modified BCA protein determination kit (SANGON Biotech, China). Carbohydrates were measured with the phenol-sulfuric acid method (Dubois et al., 1956). The total lipid content was measured as previously described (Grottoli et al., 2004). The surface area of coral was estimated indirectly by the weight of aluminum platinum paper wrapped on coral. Finally, the physiological indexes, such as symbiont cell density, biomass, protein, carbohydrates, and lipid content, were standardized by unit surface area, and the provided data were expressed as the mean ± standard error of the mean (SEM).

### DNA Extraction and Sequencing

According to the manufacturer’s instructions, total genomic DNA was extracted from 36 coral samples using a Marine Animals DNA Kit for microbial analysis. The DNeasy Plant Mini Kit was used for Symbiodiniaceae clade type determination, and DNA extraction failed from 17 samples of the T33 group. After extraction, a spectrophotometer (NanoDrop, ND2000; Thermo Fisher Scientific) was used to check the quality of the DNA samples, and a NanoDrop 2000c spectrophotometer (Thermo Fisher Scientific Inc., Waltham, United States) was used to evaluate the DNA quality. Bacterial 16S rRNA V3V4 was amplified by PCR using bacterial universal primers 338F (5′-ACTCCTACGGGAGGCAGCA-3′) and 806R (5′-GGACTACHVGGGTWTCTAAT-3′), and the internal transcribed spacer 2 region of Symbiodiniaceae nuclear ribosomal DNA was amplified using ITS specific primers ITSintfor2 (5′-GAATTGCAGAACTCCGTG-3′) and ITS2-reverse (5′-GGGATCCATATGCTTAAGTTCAGCGGGT-3′) (Grottoli et al., 2004). The PCR products were then purified with GeneJET Gel Extraction Kit (Thermo Scientific) according to the manufacturer’s instructions. Sequencing was performed on the Illumina HiSeq2500 platform. The original sequencing data were spliced and filtered, and the optimized sequence was obtained after removing the chimera according to the following criteria: (1) the reads were truncated at an average quality score < 20 over a 50-bp sliding window, (2) sequences with overlap longer than 10 bp were merged according to their overlap with an allowance of only 2-bp mismatches, and (3) the samples were separated according to the barcodes and primers (allowing only two nucleotide mismatches). The sequence was clustered and divided into the operational taxonomic units (OTUs) at a similarity level of 97%. For each representative sequence, the SIlVA (release 132, for bacteria) databases were used for classification information using the RDP classifier (Wang et al., 2007). For Symbiodiniaceae annotation, BlastN was used to select the most abundant OTU sequence as the representative sequence compared with the ITS2 database (Shi et al., 2021) as previously described (Zhu et al., 2021).

### Data Analyses

The effect of temperature on zooxanthellae density, Fv/Fm, biomass, energy substances (proteins, lipids, and carbohydrates), and growth rate was tested by one-way ANOVA, and the *post hoc* Tukey-HSD test was used to examine significant differences between the groups.

To eliminate the influence of sequencing depth, each sample of Symbiodinium and bacteria was randomly resampled according to the lowest sequencing depth and used for downstream analysis. The alpha diversity index of each sample (Shannon-Wiener Index) was calculated using the diversity function in the “vegan” package, and the results were validated using one-way ANOVA and the *post hoc* Tukey-HSD test.

Non-metric multidimensional scaling (NMDS) of the community of symbiotic algae and bacteria was performed based on the Bray–Curtis similarity, and permutational ANOVA and MANOVA (PERMANOVA) analyses were used to investigate differences in the microbial communities between groups. Taxa that differed significantly among temperatures were determined using LEfSE (linear discriminate analysis effect size) with values of linear discriminant analysis (LDA) greater than 2.

To predict the potential importance of stochastic processes on community assembly, a neutral community model (NCM) was used to determine relationships between the detection frequency of microbial taxa and their relative abundance across the wider metacommunity (Sloan et al., 2006). Calculation of 95% confidence intervals around all fitting statistics was done using 1,000 bootstrap replicates, and the parameter *R*^2^ represented the overall fit to the neutral model (Chen et al., 2019).

The nearest taxon index (NTI) was used to measure the degree of phylogenetic clustering of taxa on a single-community scale (Chen et al., 2019). To better understand the mechanism underlying the bacterial community assembly, the Stegen null model was used to evaluate the contributions of deterministic and stochastic processes to community assembly based on phylogenetic (β-nearest taxon index, βNTI) and taxonomic (Bray–Curtis–based Raup–Crick, RC-Bray) β-diversity metrics (Stegen et al., 2013). Values of | βNTI| > 2 indicate that the turnover of communities was primarily due to deterministic processes, among them βNTI < −2 represented homogeneous selection, and βNTI < −2 represented homogeneous selection (Stegen et al., 2013). Values of | βNTI| < 2 indicate stochastic processes, including homogenizing dispersal (| β-NTI| < 2 and RC-Bray < −0.95), dispersal limitation (| β-NTI| < 2 and RC-Bray > 0.95), and non-dominant processes (| β-NTI| < 2 and | RC-Bray| < 0.95) (Stegen et al., 2013).

To estimate bacterial interactions at different temperatures, OTUs present in fewer than three samples with less than 25 sequences were removed from the construction of co-occurrence networks. Robust correlations with Spearman’s correlation coefficients (ρ) > | 0.6| and Benjamini-Hochberg corrected *p*-values < 0.05 were incorporated into the network analyses in R using the “igraph” and “Hmisc” packages. The robustness of the network was completed by the provided code^1^ (Yuan et al., 2021).

All statistical analyses were performed in the R environment (v4.0.3).^2^

## Results

### Characterization of Growth and Physiological Changes

**FIGURE 2:**
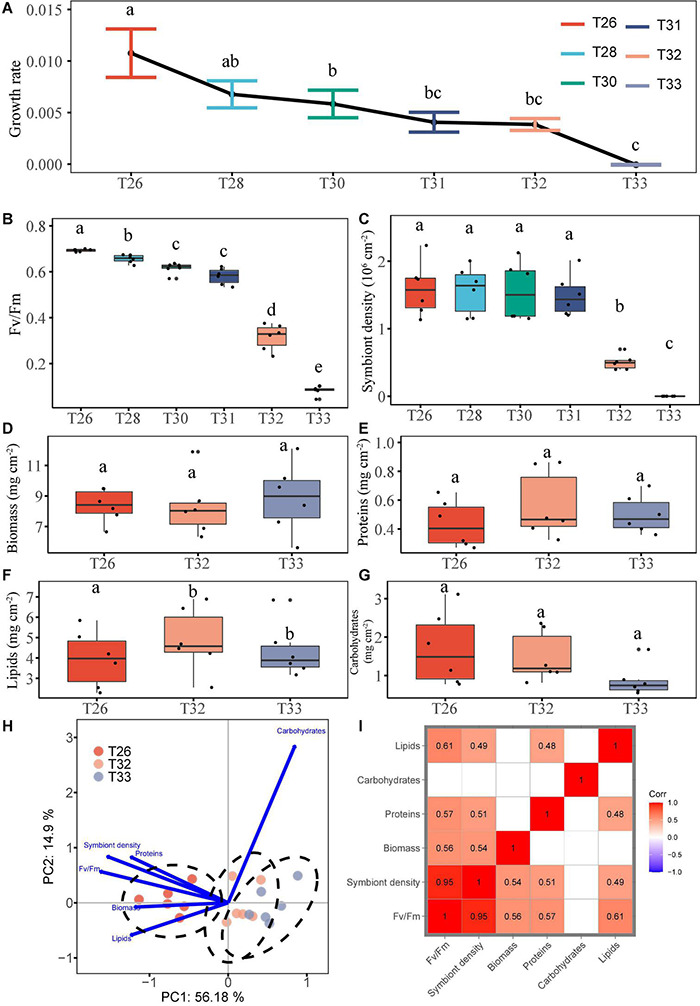
Growth and physiological parameters of coral treated with elevated temperature. **(A)** Specific growth rate. **(B)** Photosynthetic efficiency (Fv/Fm). **(C)** Symbiot density. **(D)** Biomass. **(E)** Protein. **(F)** Lipid. **(G)** Carbohydrate. **(H)** PCA. **(I)** Correlation analysis. Data are expressed as mean ± SEM (*n* = 6). Different lowercase letters denote significant differences from different treatments (*P* < 0.05).

During the entire experiment, the specific growth rate of coral (μ, *n* = 6; mean ± SEM) was significantly decreased when the temperature was increased from 26°C (μ = 0.01 ± 0.002) to 30°C (μ = 0.006 ± 0.001; *P* < 0.05), and the coral almost stopped growing at 33°C (μ was approximately equal to 0; Figure 2A and Supplementary Table S1). Meanwhile, we found that the Fv/Fm was significantly decreased with increasing temperature. In particular, it was less than 0.5 in the T32 (0.32 ± 0.02) and T33 groups (0.08 ± 0.001), which was decreased by 54 and 88% compared with the T26 group, respectively (Figure 2B). The zooxanthellae density was 1.59 ± 0.15 10^6^ cells/cm^2^ at 26°C at the beginning of the experiment, and it remained stable during the middle stage when the temperature was increased to 31°C (*P* > 0.05). The zooxanthellae density was decreased by 68% after the temperature reached 32°C (0.50 ± 0.04 10^6^ cells/cm^2^). Especially, coral bleaching was very obvious during the T33 stage with the zooxanthellae density of almost 0 (Figure 2C), whereas no coral death was observed during the experiment. Results of one-way ANOVA showed that the biomass (Figure 2D; *P* = 0.069), protein (Figure 2E; *P* = 0.055), and carbohydrate contents (Figure 2G; *P* > 0.05) of coral had no significant changes among the three groups, and only the lipid content of the T32 and T33 groups was much lower compared with the T26 group (Figure 2F; *P* < 0.05). However, the biomass, protein, and lipid contents of the T33 group were lower compared with the T26 group, and the average contents were decreased by 22.60, 34.25, and 37.28%, respectively. In contrast, the carbohydrate content was increased with increasing temperature although there was no significant difference (*P* > 0.05). We further used PCA to provide overall visualization of the physiological indexes of corals at three temperatures (Figure 2H). The results show that the photosynthetic physiological indexes of coral under T33 and T32 were significantly different from those of the T26 group (*P* < 0.05). In the combined measurements of these three temperatures, we found more positive correlations between paired physiological indicators (Figure 2I). There was a significant positive correlation between photosynthetic physiological indexes (Fv/Fm, symbiotic density) and biomass or two energy substances (protein and lipid; *P* < 0.05). Only a significant correlation was found between protein and lipid contents (*P* < 0.05), and there was no correlation between carbohydrates and other indexes.

### Changes of Symbiodiniaceae Diversity and Community

**FIGURE 3:**
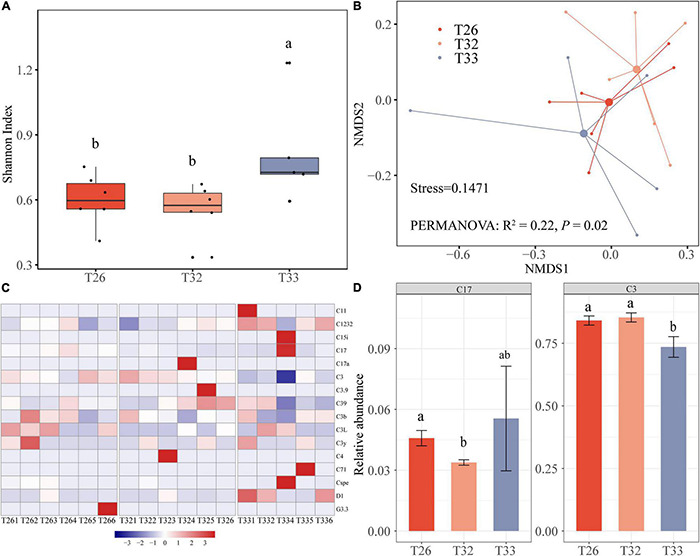
The diversity and the community of Symbiodiniaceae. **(A)** Shannon index of bacteria. **(B)** NMDS analysis performed on community composition dissimilarities (Bray-Curtis) across different temperatures. **(C)** Symbiodiniaceae composition. **(D)** Dominant Symbiodiniaceae with a difference at different temperatures.

For 17 coral samples, a total of 657,413 Symbiodinium ITS2 sequences were retained after quality control with an average of 46,958 Symbiodinium ITS2 sequences per coral sample. After subsampling at the same sequencing depth, 45 OTUs at a similarity threshold of 97% were obtained. The rarefaction curves indicate that the number of Symbiodinium ITS2 sequences could meet the needs of the current diversity analysis. We observed that the Shannon diversity index of Symbiodiniaceae in the T33 group (0.81 ± 0.11) was significantly higher compared with the T26 and T32 groups (Figure 3A; *P* < 0.05). The NMDS analysis revealed a division of the Symbiodiniaceae community in the different treatments (Figure 3B), and the PERMANOVA test indicated that the composition of the Symbiodiniaceae community was different at different temperatures (*R*^2^ = 0.22, *P* = 0.02). There was no significant difference in the Symbiodiniaceae structure between T26 and T32 (*R*^2^ = 0.04, *P* > 0.05), whereas the difference between T33 and T26 tended to be significant (*R*^2^ = 0.16, *P* = 0.051). In particular, there was a significant difference in the zooxanthellae structure between T32 and T32 (*R*^2^ = 0.21, *P* = 0.013). Most OTUs of Symbiodinium ITS2 sequences were assigned to Symbiodinium subclade C3, C1232, and C17, which covered more than 96% of Symbiodinium ITS2 sequences. Symbiodinium subclade C3 dominated in every coral with a relative abundance of 58.07–93.10%, and the relative abundance in T33 was significantly lower compared with T26 and T32 (*P* < 0.05). The relative abundance of C1232 fluctuated little, whereas the relative abundance of C17 in T32 was significantly lower compared with T26 and T33 (Figure 3C). In addition, we found that some scarce species were increased or decreased in individuals in different groups, such as the increased relative abundances of C11, C15i, and D1 in individual samples of T33 (Figure 3D). The changes in diversity and community structure of Symbiodiniaceae were mainly caused by the changes in the relative abundance of predominant subclade and “Symbiodiniaceae rare biosphere.”

### Changes in Coral-Associated Bacterial Community

**FIGURE 4:**
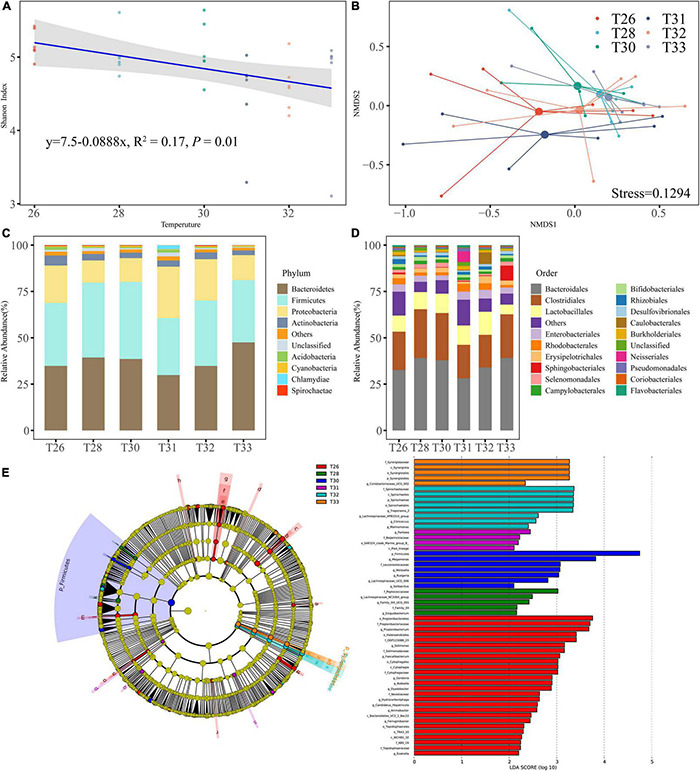
The diversity and the community of coral-associated bacteria. **(A)** Shannon index of bacteria. **(B)** NMDS analysis performed at different temperatures on community composition dissimilarities (Bray-Curtis). **(C)** Microbial community composition of bacterial phyla in all samples. **(D)** Microbial community composition of bacterial order in all samples. **(E)** Indicator microbial groups at each treatment with the LDAvalues higher than 2.0.

A total of 36 coral samples were analyzed in the present study for bacteria, and 1,802,874 raw reads were obtained, of which 1,724,741 high-quality sequences were successfully classified and passed quality filtration. Moreover, 3,976 bacterial OTUs were clustered at a minimum sequence similarity of 97%, and rarefaction curves indicated that most of the diversity could be covered by the resampling depth of 24,434 reads. The bacterial Shannon diversity index was only significantly different between T26 and T31 (Figure 4A), and there was no significant difference between other temperatures (*P* > 0.05), whereas it tended to become smaller as the temperature was increased (*R*^2^ = 0.17, *P* = 0.01). NMDS based on the Bray–Curtis distance indicated no significantly different division between the coral bacterial communities exposed to different temperatures, which closely corresponded to the results of PERMANOVA (Figure 4B; *R*^2^ = 0.16, *P* > 0.05). Compared with the control at 26°C, short-term exposure to high temperatures did not seem to cause any large-scale changes in the structure of the coral bacterial community. Bacteroidetes and Firmicutes were the most abundant phyla (average relative abundances were 37.50 and 35.93%, respectively), followed by Proteobacteria (18.09%) and Actinobacteria (3.57%). The remaining 4.91% of the sequence reads predominantly consisted of Actinobacteria, Acidobacteria, Cyanobacteria, Chlamydiae, Spirochaetae, and another rare phylum (Figure 4C). Most OTUs in Bacteroidetes were assigned to Bacteroidales (35.12%), and the most abundant categories in Firmicutes were ranked as follows: Clostridiales (21.98%), Lactobacillales (9.42%), and Erysipelotrichales (2.41%). The relative abundances of other dominant orders Sphingobacteria, Selenium, Campylobacteria, Bifidobacteria, Rhizobiales, and Desulfovibrionales ranged from 0.58 to 1.69% (Figure 4D). LEfSe scores were computed for taxa differentially abundant across different temperature treatments (Figure 4E). The results showed that most indicator bacteria were mainly enriched in T26, including three classes, five orders, seven families, and 11 genera categories. Species of two families and two genera categories were more representative in T28. Firmicutes was the main discriminant category of T30, and the five genera from Leuconostocaceae were enriched in T31. Spirochaetae included one class, one order, and one family, and four genera had significantly higher abundance in T32, and bacteria from Synergistetes was a significantly related indicator in T33.

### Assembly Processes of Coral-Associated Bacterial Community

**FIGURE 5:**
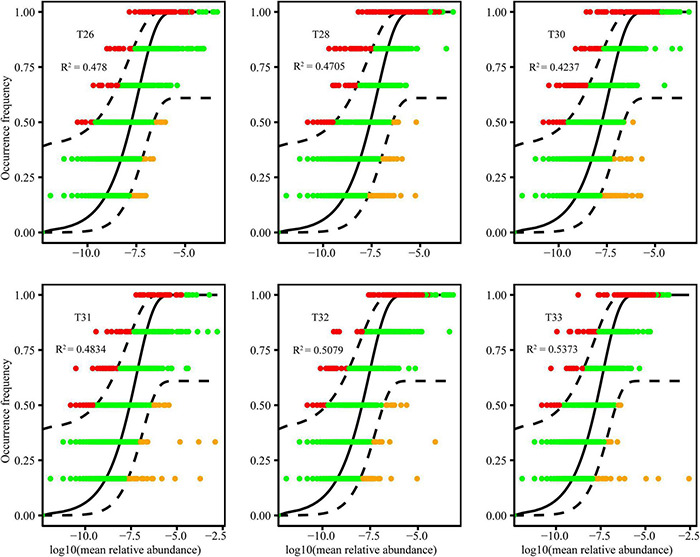
The fit of the NCM of coral-associated bacterial community assembly at different temperatures. The blank lines indicate the best fit to the NCM, and the dashed lines represent 95% confidence intervals around the model prediction. *R*^2^ indicates the goodness of fit to this model.

We fitted the bacterial community to the NCM, which successfully estimated most of the relationship between the frequency of OTUs and the change in their relative abundance (Figure 5). The goodness of fit of bacteria at 28–30°C (community-explained variances ranged from 42.37 to 47.80%) was generally lower than 31–33°C (community-explained variances ranged from 48.34 to 58.43%). Furthermore, the goodness of fit of the neutral model in the bacterial community of T31–33 was primarily improved, and the relative contribution of the stochastic process was gradually increased with increasing temperature. The results show that the NCM better described the relationship between the frequency of OTUs and their relative abundance. The stochastic process was essential for shaping the community assembly of coral symbiotic bacteria.

**FIGURE 6:**
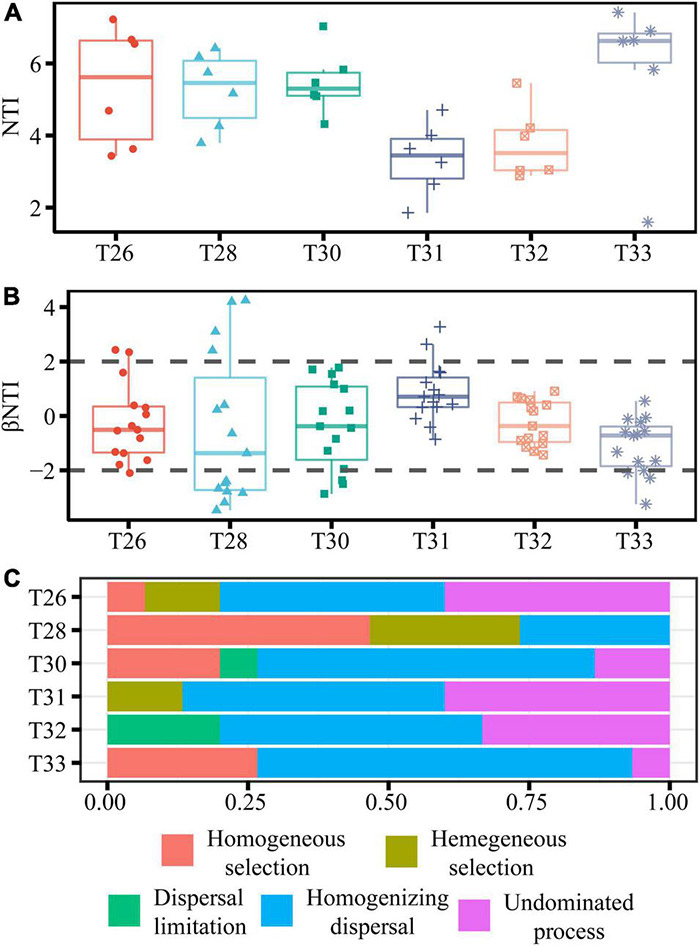
Relative influences of deterministic and stochastic processes on community assembly. **(A,B)** Are the boxplots of the NTI and βNTI for all pairs of communities within coral samples at different temperatures. **(C)** The percentage of turnover in community assembly governed primarily by various deterministic and stochastic processes.

The most significant mean NTI for symbiotic bacteria was found in T33, and it was more than zero (indicating phylogenetic clustering) in all temperature treatments (Figure 6A). βNTI values for the coral-associated bacterial community were more than 2 in T28, and most βNTI values in other temperature treatments ranged from −2 to + 2 (Figure 6B). Based on the Stegen null model, RC-Bray and βNTI were used to quantify the determinism and stochasticity of microbial community assembly (Figure 6C). Stochastic (80%) assembly processes governed the bacterial community in T26. The bacterial community in T28 was mainly dominated by homogeneous (46.67%) and heterogeneous selection (26.67%), and the contribution of the stochastic assembly was only 20%. However, the community in T30 was mainly driven by the stochastic assembly (80%), and the deterministic contribution was only 20%. The stochastic processes (dispersal limitation, homogenizing dispersal, and undominated processes) accounted for 87, 100, and 73% of the community assembly processes in T31–33, respectively. Overall, we further confirmed that the community assembly of the coral-associated bacterial community was primarily controlled by the stochastic process when the temperature was increased.

### Interaction Network and Co-occurrence Network Stability of Microbial Community

**FIGURE 7:**
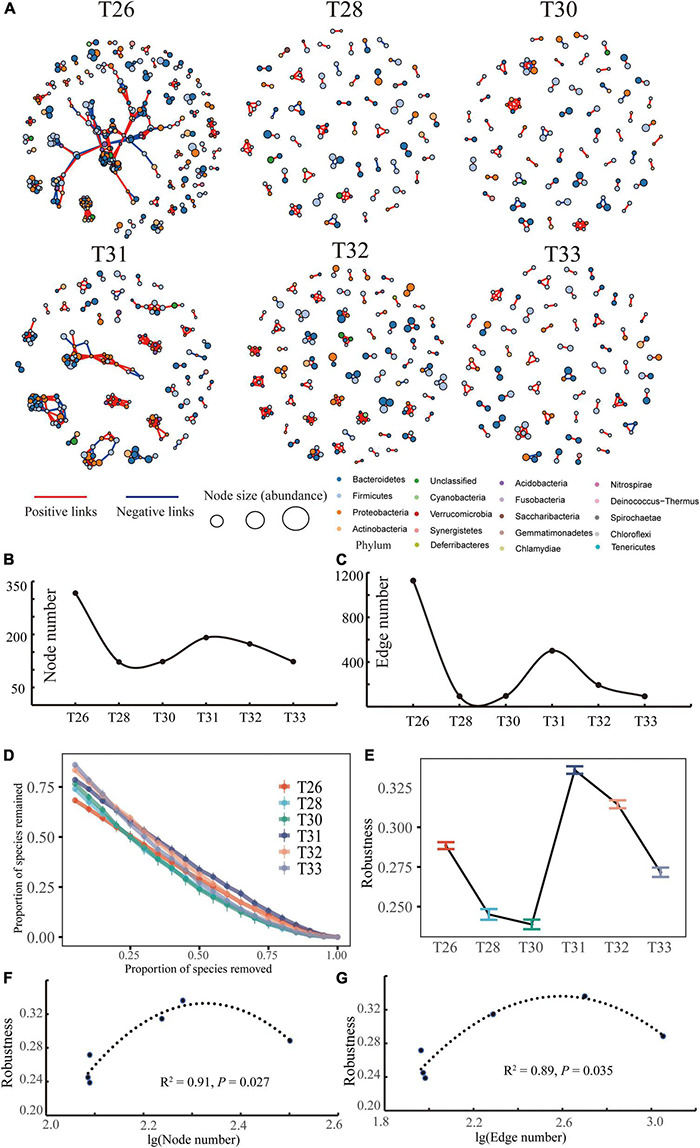
Co-occurrence patterns of the microbial community. **(A)** Microbial co-occurrence networks across the different treatments. The nodes are colored based on the phylum level for coral-associated bacteria. The size of each node is proportional to the relative abundance of OTUs; the thickness of the edge is proportional to the value of Spearman’s correlation coefficient. **(B,C)** Are the node number and edge number of the co-occurrence networks at the different temperatures, respectively. **(D,E)** Are network stability and the robustness (the proportion of taxa remained with 50% of the taxa randomly removed from each co-occurrence network) of networks under different temperature conditions. **(F,G)** Are the relationship between network robustness and microbial network complexity (node number and edge number).

Microbial co-occurrence networks were constructed to estimate species coexistence at different temperatures (Figure 7A). The microbiome of the T26 group formed a more extensive network with more nodes (317) and connections (1,129), which were more than the network of the T28 group (122 edges among 94 nodes) and T30 group (123 edges among 96 nodes). Interestingly, the bacterial symbiosis network in the T31 group was composed of 191 nodes and connected by 501 edges. T32 formed smaller networks with fewer nodes and fewer connections (173 edges among 123 nodes), and the network of the T33 group contained 123 edges and 92 nodes. The total number of nodes and the degree of connection of the interaction network indicated that the bacterial networks were more complex in T26 and T31 (Figures 7B,C). The bacterial network at each temperature had more positive correlations than negative correlations (65.54, 82.98, 80.21, 79.84, 90.21, and 92.39%, respectively), implying that there were potential cooperation and beneficial relationships between coral-associated bacteria. There were relatively more negative correlations in T26 and T31, indicating a specific competitive relationship between their bacterial species. In addition, we compared network stability between different temperatures based on the network robustness (Figures 7D,E). The stability of the coral-associated bacterial network was sharply decreased when the temperature was increased from 26°C to 30, whereas it quickly established high stability. Robustness was the highest in T31, and the network robustness of the T30 group was the lowest. Finally, we found that the network robustness and complexity, such as nodes and links, showed a significantly inverted U-shaped relationship (Figures 7F,G).

## Discussion

### High Temperature Affects the Physiological Characterization, Diversity, and Community Composition of Symbiodiniaceae Symbionts

The decrease of Fv/Fm during the stress (for example, the increase of SST) can be used to determine the damage to the PSII of zooxanthellae, and the downregulation of PSII photochemistry may have a photoprotective effect (Bhagooli and Hidaka, 2004; Rosset et al., 2017). We observed that the Fv/Fm of *A. hyacinthus* was significantly decreased with increasing temperature, indicating that the photosynthetic function in zooxanthellae was inhibited. Especially in the T32 and T33 stages, the Fv/Fm was less than 0.5, which was related to low light utilization, low symbiotic density, or damage of PSII (Mueller and Schupp, 2020). In addition, the density of zooxanthellae is an essential index of coral in response to heat stress (Wooldridge, and Scott, 2014). The density of zooxanthellae of coral remained stable in the middle stage from 26 to 31°C (2°C higher than the local long-term summer average and 1°C higher than the local bleaching threshold) (Jiang et al., 2017), indicating that *A. hyacinthus* had specific resistance in a short time at 31°C. Especially in the T33 stage, coral bleaching was very obvious, and bare bones were observed (but the coral did not die and even survived for 2 months). In the last stage of heat treatment, the symbiotic relationship between corals and their photosynthetic symbionts collapsed due to heat, corals released their symbionts, the number of flagellates was decreased rapidly, and corals became bleached. Bleaching sensitivity and the function of the symbiotic relationship depend on the symbiotic density and photosynthetic capacity (Xu et al., 2017). Before symbiotic decomposition, the decrease of Fv/Fm is often observed in coral, and the photosynthetic performance is sharply decreased with the decrease of zooxanthellae density (Bessell-Browne et al., 2017).

When coral lose algal symbionts, photosynthesis is decreased, indicating that the amount of fixed carbon transferred from zooxanthellae to hosts is sharply decreased (Davy et al., 2012; Jung et al., 2021). To offset the reduction of photosynthetic products, coral can meet their daily metabolic energy needs by catabolizing stored energy reserves (such as lipids, proteins, and carbohydrates), increasing feeding rate, decreasing metabolic rates, and reducing calcification rate (Baumann et al., 2014). Some studies find that the energy reserves and tissue biomass of some coral remains unchanged during bleaching, which may be related to the ability to use heterotrophic fixation of carbon sources to meet their metabolic needs (Wall et al., 2019; Zhu et al., 2021). However, *Porites compressa* cannot increase feeding when bleached, relying completely on energy reserves after bleaching (Levas et al., 2013). In the present study, the carbohydrate content remained unchanged throughout the bleaching stage, which was inconsistent with the preference of bleached corals in the subtidal zone for catabolizing energy-poor carbohydrates during peak bleaching (Levas et al., 2013). However, we observed a 28% decline in protein concentrations in T32 and a 37% decline in lipid stores in T33 compared with T26. Although there was a certain number of zooxanthellae in coral at 32°C, the photosynthetic capacity was too low due to the damage of PSII. There was a significant correlation between protein and lipid contents (*P* < 0.05), and the decrease in proteins of the T33 group might be related to the conversion to lipids. Generally, the maintenance of lipid stores is the primary source of nutrition during bleaching due to their high energetic value compared with proteins and carbohydrates (Grottoli et al., 2004). In the process of heat stress, *A. hyacinthus* reduced energy reserves (mainly lipids and proteins) due to coral tissue loss and the decline of the density and photochemical efficiency of zooxanthellae, resulting in the decreased biomass and the stagnation of the coral growth rate, which might eventually lead to coral death.

The “adaptive bleaching hypothesis” states that coral adapts to elevated temperatures by shuffling or switching symbionts (Baker et al., 2004). In addition, coral can increase adaptability to climate change by increasing the diversity of symbiotic families (Zhenjun et al., 2019). However, a previous study shows that the composition and diversity of the *Porites lutea* Symbiodiniaceae community remain unchanged throughout the bleaching event, indicating that the switching and/or shuffling of Symbiodiniaceae types may not be the primary mechanism in response to increased SST (Pootakham et al., 2018). In the present study, we observed that the Shannon diversity index of Symbiodinium subclades was changed significantly at 33°C, and the bleaching event significantly changed the community composition. This finding is consistent with the “adaptive bleaching hypothesis” and provides preliminary evidence. The coral host usually consists of high abundance “dominant types” or the very low abundance “Symbiodinium rare biosphere” (Pootakham et al., 2018).

Many types of Symbiodinium ITS2 sequences were identified in the present study. Most OTUs were assigned to Symbiodinium subclade C3, C1232, and C17, covering more than 96% of Symbiodinium ITS2 sequences. However, most of them belonged to rare background types, representing a low-abundance, high-diversity group (Ziegler et al., 2018). Although type C3 Symbiodinium is generally considered a worldwide and thermally sensitive generalist, the prevalence found in heat stress–tolerant corals from Abu Dhabi may imply that this type of phenotypic plasticity is much greater than previously thought (Hume et al., 2013). We found that subclade C3 dominated each coral sample, and the relative abundance was significantly reduced in T33. The rare background types are also crucial for adapting coral symbionts to heat stress (Fabina et al., 2013; Ziegler et al., 2018). Some coral species can adapt to thermal conditions by changing the type and relative abundance of the heat-resistant background types (Ziegler et al., 2018). This study finds that the abundance of some scarce heat-resistant species were increased in bleaching *A. hyacinthus*. For example, the abundances of Symbiodiniaceae type D1 and C151, which are considered heat-resistant species, were increased in individual samples of T33. This finding indicates that *A. hyacinthus* enhances the resistance to bleaching without changing the composition of the symbiotic clade, which might be related to the longer transplantation time (12–27 months) or the more significant temperature fluctuations (29–35°C) in their research (Ziegler et al., 2017). Our results indicate that bleaching *A. hyacinthus* could increase the proportion of diversity and the rare heat-resistant species under thermal stress and reduce the relative abundance of thermally sensitive types of Symbiodiniaceae rather than the uptake of new symbiont types (Thomas et al., 2019). In addition, the loss of coral tissue might also lead to changes in the diversity and community composition of zooxanthellae measured by high-throughput sequencing. However, we believe that this unstable symbiont community composition did not have a good effect when considering the sharp decrease of zooxanthellae density and Fv/Fm observed in the above results.

### Diversity of the Associated Bacteria Is Reduced, but the Bacterial Structure Is Stable and Has a Complex Relationship With Bleaching Phenotypic Response

γ- and α-proteo bacteria usually dominate the bacterial communities of coral and other abundant bacteria, including Bacteriodetes, Firmicutes, Actinobacteria, and Cyanobacteria (Hernandez-Agreda et al., 2017). Most OTUs in Bacteroidetes were allocated to Bacteroidales (35.12%), showing that Bacillus could decompose organic matter and might participate in the metabolism of nitrate (Zhu et al., 2018). The most abundant Firmicutes were Clostridiales (21.98%), and it is found that their relative abundance in the host is increased under heat stress, which is a highly polyphyletic group of Firmicutes (Yu et al., 2020). Studies show that the number of Bacteroides, Actinomycetes, and Firmicutes is increased when *Pseudoplexaura crucis* is exposed to elevated temperature and/or UVR, which may be related to the fact that these bacterial phyla may participate in the nutrient cycle and may not be detrimental to the host (McCauley et al., 2020). We find some confusing functional descriptions about coral-associated bacteria. For example, Rhodobacterales represent a very diverse group with heterotrophic and phototrophic members and may play an essential role in the heat resistance of coral (McCauley et al., 2020). However, they are described as putative opportunistic microorganisms and generally enriched in diseased coral in other literature (Roder et al., 2014; Zaneveld et al., 2016). In the present study, we detected various bacterial groups involved in nitrogen fixation, energy metabolism, and heat resistance. However, their specific roles and the complete functional complexity of coral-related microbial communities have not been fully determined and must be verified in the future.

When coral are exposed to potential stressors (such as high temperature), a decrease in beneficial bacteria may occur, and at the same time, opportunistic and potentially harmful bacteria may increase, leading to a harmful increase in the diversity of bacterial combinations (Roder et al., 2014; Zaneveld et al., 2016). For example, after coral are exposed to elevated ammonium stress (Zhang et al., 2021), reduced pH (Meron et al., 2011), and thermal stress (Meron et al., 2011), the related bacterial diversity is generally increased, and the relative abundance of bacteria related to diseased and stressed coral, such as Vibrioceae, Alphaproteobacteria, Rhizobiales, Rhodobacteriales, Caulobacteriales, and Rhodospillales, is increased, finally leading to the increased diversity of coral-related microorganisms (Meron et al., 2011). In another study, there is no significant difference in OTU diversity between healthy and affected coral, whereas significant shifts of Alphaproteobacteria, Betaphaproteobacteria, and Gammaproteobacteria are also observed in affected corals (Cárdenas et al., 2012). In our current study, the sequence ratios of taxa, usually pathogenic or opportunistic bacteria (such as Vibrionales, Alteromonadales, and Flavobacteriales), remained unchanged. Furthermore, the confirmed coral pathogens were not found in this study, indicating that thermally stressed *Acropora hyacinthus* were unlikely to be threatened by these common bacterial diseases. Therefore, although the bacterial diversity tended to decrease with increasing temperature, this was attributed to the adjustment of the primary community composition unique to each coral species rather than the influence of disease. Furthermore, analysis in response to changes in coral bacterial diversity under stress should be combined with changes in the abundance of specific bacterial members.

Environmental stress factors can destroy beneficial microbes and promote the invasion of microbes in the surrounding environment. Moreover, we have not observed an increase in the relative abundance of Vibrio or other known coral pathogen species when the SST is increased (Cárdenas et al., 2012). The small changes in the abundance of bacterial communities may be mediated through host-mediated interactions or plasticity of the microbial community (van de Water et al., 2018). For example, Firmicutes was the main distinguishing category of T30, and it is reported that the abundance of Firmicutes in coral exposed to various stress conditions will increase (Garren et al., 2009). However, the critical coral microbial communities in this study were hardly changed although the abundance of some bacteria was altered. This finding was different from the stable symbiotic bacterial community structure of bleached corals, which may seem to be different in the overall metabolic state of the coral holobiont and help cope with heat stress (Gajigan et al., 2017; Zhu et al., 2021). A previous study reports that the relevant bacterial community in *Pocillopora verrucosa* remains very stable under the conditions of coral bleaching and severe tissue sloughing (> 90% tissue loss resulting in host mortality) (Pogoreutz et al., 2018). This study finds that the visual appearance of coral, zooxanthellae density, and photosynthetic efficiency provide clear evidence of tissue bleaching during heat stress, especially at the threshold from 32 to 33°C. At the same time, these small OTU changes (as revealed by the LEfSe analysis) did not indicate that the adaptation response could confer probiotics or protection to cope with rising temperatures. The coral hosts with a “stable” (and possibly strongly selected) microbiome observed in *P. verrucosa* may have a highly uneven bacterial community (Pogoreutz et al., 2018), whereas our results find a more complex and classified bacterial composition. Therefore, the relationship between changes in bacterial diversity and community composition or coral health might be more complicated than imagined, indicating that the specific relationship between microbiome structure flexibility and coral host physiology still requires in-depth correlation research.

### Coral-Associated Bacterial Community Assembly Is Mainly Shaped by Stochastic Process and Species Coexistence Patterns

The positive NTI values in our study suggest that the communities were more phylogenetically clustered across the overall phylogeny than expected by chance, reflecting the importance of habitat filtering (in which a group of closely related species shares a characteristic, or suite of traits, which enables them to persist in a given habitat) (Horner-Devine and Bohannan, 2006; Pontarp et al., 2012). The coral host is a unique habitat for the microorganisms in the symbiosis. The microorganisms are filtered to maintain the native microbiota composition unique to each coral species, suggesting a dominant role for the coral host in structuring the microbiome (Dunphy et al., 2019). In addition, the higher phylogenetic clustering may be related to the successful establishment and maintenance of specific coral–bacterial associations because members of coral-associated prokaryotes may resist pathogenic microbes by preventing their colonization through the physical occupation of otherwise available niches (Pootakham et al., 2018), which is consistent with the diversity changes rather than a reflection of disease status in thermally stressed *A. hyacinthus*. There was no significant relationship between NTI and temperature in our study, indicating that the increase in temperature did not change the degree of phylogenetic clustering in the microbial community. Long-term culture history can actively select microorganisms with adaptive advantages under relatively constant environmental conditions, thereby reducing the impact of ecological filtration (Grottoli et al., 2018; Guo et al., 2018). Therefore, the experimental species *A. hyacinthus* in this study survived after stress (because the field observation revealed that extensive albinism was found in March). This finding was consistent with the results that the *A. hyacinthus* from the highly variable warm environment often exposed to heat stress maintains its primitive bacterial community composition during the whole process of heat stress (Ziegler et al., 2017).

The construction of community composition is different from the level of control a host exerts over the composition of its microbiome and different taxa within the microbiome (Adair and Douglas, 2017). Variable selection (such as the selective pressure resulting from environmental conditions) is the main factor determining the compositional change in the coral reef–associated bacterial communities across the 2,000-km spread of the Red Sea according to Stegen’s ecological modeling framework (Pearman et al., 2019), whereas stochastic processes are found to be more common in bacterial community assembly in healthy *Porites compressa* and *Pocillopora meandrinan* in another study (Price et al., 2021). NCM explains 48.34–58.43% of the community variance in different temperature treatments in our current study. Because the NCM did not explain 100% of the community assembly, we further used βNTI and RC-Bray based on the Stegen null model to explore the relative roles of stochastic and deterministic processes in shaping the assembly. However, results from the null and neutral theory–based process models collectively support that the stochastic process (variable selection) played a more critical role in bacterial community assembly of thermally stressed *A. hyacinthus*, and the weak effect of temperature on microbial community structure and assembly could be explained by the relative importance of stochastic processes. A potential explanation is that coral hosts could be unique habitats resulting in less environmental filtering, and in coral with species pools characterized by environmental generalist prevailing bacterial taxa that are well adapted to high temperatures, the advantage of the stochastic process occurs and overwhelms the deterministic process (Wang et al., 2013; Jiao et al., 2020). In addition, the stochastic process shows that, when the competitive abilities of species closely match, or random changes are not related to environmental adaptability, species can co-occur with fairly overlapping niches (Wang et al., 2013; Jiao et al., 2020).

Microorganisms occupying specific niches *via* horizontal gene transfer can form complex interaction networks. Determining the link between community assembly and species coexistence is critical for understanding the mechanisms supporting community diversity (Jiao et al., 2020). For example, negative biological interactions have essential contributions in shaping the assembly of soil microbial communities (Romdhane et al., 2021). However, network topology parameters consistently indicate that the bacterial co-occurrence network fluctuates at different temperatures and became more straightforward with increasing temperature. We found potential cooperation and mutual benefits between bacterial communities, which might cooperate more to adapt to high temperatures or in similar niches, and these prokaryotes are less affected by warming. Stress conditions that reduce the ability of the host or its microbiota to regulate community composition may transform the coral microbiome into a pathogen-dominated stable state, leading to the transformation of the microbiome network into an unstable state (Mao-Jones et al., 2010; Zaneveld et al., 2017). However, our results reveal that the stability of the coral bacterial network decreased sharply with increasing temperature, whereas a high degree of stability was rapidly established. In addition, there was a significantly inverted “U” relationship between network robustness and network complexity, which was inconsistent with the central ecological view that network stability is often closely positively relevant to network complexity (Mao-Jones et al., 2010; Zaneveld et al., 2017). The explanation might be related to the interference affecting the cooperation and reducing competition between bacterial communities, thus increasing the influence of stochastic processes in community assembly (Jiao et al., 2021; Yun et al., 2021). The temperature might cause microbial co-occurrence patterns to be less complex but enhance the stability of the interaction between bacteria, whereas this effect was not a non-linear response. Moreover, competition with more species reduces diversity, whereas randomness becomes more critical as resource availability increases (Chase, 2010). Therefore, the dominance of the stochastic assembly process may indicate that microbial groups coexist more frequently because microbial coexistence is more common under weak environmental filtration due to the advantage of the stochastic process. In our present study, this finding suggests that bacterial interaction as a selective force could contribute to the assembly of coral symbiotic bacterial communities at different temperatures.

## Conclusion

This study holistically reveals the physiological response and community dynamics of holobionts in thermally stressed *A. hyacinthus*. Our data explains the community assembly and species coexistence mechanism of symbiotic bacteria. The symbiotic relationship between *A. hyacinthus* and their photosynthetic symbionts was decomposed when reaching the bleaching threshold. Bleaching coral with loss of algal symbionts mainly catabolized lipids and proteins to maintain survival, resulting in reduced tissue biomass and growth. Coral might improve the diversity of Symbiodiniaceae and change the community structure by reducing the relative abundance of thermally sensitive generalists and increasing rare heat-resistant species. Bacterial diversity would be decreased with increasing temperature. At the same time, there was little change in a vital coral microbial community, which was the adjustment of bacterial community composition rather than the response of pathogenic or opportunistic bacterial invasion. The stochastic process was essential for shaping the community assembly of coral symbiotic bacteria. Therefore, the temperature had a weak impact on the bacterial community structure and assembly, and bleaching coral always maintained the original community membership and abundance under thermal stress. Although the network structure fluctuated and tended to be simplified with increasing temperature, the coral bacterial symbiotic network would quickly establish a high degree of stability. Stochastic processes and network interaction would help the bacterial community cooperate more to adapt to high-temperature niches, which would help thermally stressed *A. hyacinthus* maintain the coexistence and stability of native bacteria in response to elevated temperatures.

## Data Availability Statement

The datasets presented in this study can be found in online repositories. The names of the repository/repositories and accession number(s) can be found below: https://www.ncbi.nlm.nih.gov/, PRJNA787103 and https://www.ncbi.nlm.nih.gov/, PRJNA787317.

## Author Contributions

WZ and XL conceived the study. WZ performed the analyses, drew all pictures, and drafted the manuscript. XBL, MZ, XKL, HY, and JH performed the research. AW contributed to supervision and funding acquisition. XL framed the manuscript and contributed to revisions. All authors reviewed the manuscript.

## Conflict of Interest

The authors declare that the research was conducted in the absence of any commercial or financial relationships that could be construed as a potential conflict of interest.

## Publisher’s Note

All claims expressed in this article are solely those of the authors and do not necessarily represent those of their affiliated organizations, or those of the publisher, the editors and the reviewers. Any product that may be evaluated in this article, or claim that may be made by its manufacturer, is not guaranteed or endorsed by the publisher.

## Footnotes

1
https://github.com/Mengting-Maggie-Yuan/warming-network-complexity-stability

2
http://www.r-project.org/

## Supplementary Material

Supplementary Table 1 Specific growth rate of corals in elevated temperature.

## References

[B1] Adair K. L. Douglas A. E. (2017). Making a microbiome: the many determinants of host-associated microbial community composition. *Curr. Opin. Microbiol.* 35 23–29. 10.1016/j.mib.2016.11.002 27907842

[B2] Baker A. C. Starger C. J. McClanahan T. R. Glynn P. W. (2004). Corals’ adaptive response to climate change. *Nature* 430:741. 10.1038/430741a 15306799

[B3] Barshis D. J. Ladner J. T. Oliver T. A. Seneca F. O. Traylor-Knowles N. Palumbi S. R. (2013). Genomic basis for coral resilience to climate change. *Proc. Nat. Acad. Sci. USA* 110 1387–1392. 10.1073/pnas.1210224110 23297204 PMC3557039

[B4] Baumann J. Grottoli A. G. Hughes A. D. Matsui Y. (2014). Photoautotrophic and heterotrophic carbon in bleached and non-bleached coral lipid acquisition and storage. *J. Exp. Mar. Biol. Ecol.* 461 469–478. 10.1016/j.jembe.2014.09.017

[B5] Bessell-Browne P. Negri A. P. Fisher R. Clode P. L. Duckworth A. Jones R. (2017). Impacts of turbidity on corals: the relative importance of light limitation and suspended sediments. *Mar. Pollut. Bull.* 117 161–170. 10.1016/j.marpolbul.2017.01.050 28162249

[B6] Bhagooli R. Hidaka M. (2004). Photoinhibition, bleaching susceptibility and mortality in two scleractinian corals, platygyra ryukyuensis and stylophora pistillata, in response to thermal and light stresses. *Comp. Biochem. Physiol. Part A: Mol. Integ. Physiol.* 137 547–555. 10.1016/j.cbpb.2003.11.008 15123191

[B7] Buerger P. Alvarez-Roa C. Coppin C. W. Pearce S. L. Chakravarti L. J. Oakeshott J. G. (2020). Heat-evolved microalgal symbionts increase coral bleaching tolerance. *Sci. Adv.* 6:a2498. 10.1126/sciadv.aba2498 32426508 PMC7220355

[B8] Burns A. R. Stephens W. Z. Stagaman K. Wong S. Rawls J. F. Guillemin K. (2016). Contribution of neutral processes to the assembly of gut microbial communities in the zebrafish over host development. *ISME J.* 10 655–664. 10.1038/ismej.2015.142 26296066 PMC4817674

[B9] Cárdenas A. Rodriguez-R L. M. Pizarro V. Cadavid L. F. Arévalo-Ferro C. (2012). Shifts in bacterial communities of two caribbean reef-building coral species affected by white plague disease. *ISME J.* 6 502–512. 10.1038/ismej.2011.123 21955993 PMC3280130

[B10] Chase J. M. (2010). Stochastic community assembly causes higher biodiversity in more productive environments. *Science* 328 1388–1391. 10.1126/science.1187820 20508088

[B11] Chen W. Ren K. Isabwe A. Chen H. Liu M. Yang J. (2019). Stochastic processes shape microeukaryotic community assembly in a subtropical river across wet and dry seasons. *Microbiome* 7:138. 10.1186/s40168-019-0749-8 PMC6806580 31640783

[B12] Conti-Jerpe I. E. Thompson P. D. Wong C. W. M. Oliveira N. L. Duprey N. N. Moynihan M. A. (2020). Trophic strategy and bleaching resistance in reef-building corals. *Sci. Adv.* 6:z5443. 10.1126/sciadv.aaz5443 32300659 PMC7148090

[B13] Davy S. K. Allemand D. Weis V. M. (2012). Cell biology of cnidarian-dinoflagellate symbiosis. *Microbiol. Mol. Biol. Rev. Mmbr* 76 229–261. 10.1128/MMBR.05014-11 22688813 PMC3372257

[B14] Dini-Andreote F. Stegen J. C. Elsas J. V. Salles J. F. O. (2015). Disentangling mechanisms that mediate the balance between stochastic and deterministic processes in microbial succession. *Proc. Nat. Acad. Sci.* 2015:112. 10.1073/pnas.1414261112 25733885 PMC4371938

[B15] Dubois M. Gilles K. A. Hamilton J. K. Rebers P. A. Smith F. (1956). Colorimetric method for determination of sugars and related substances. *Analy. Chem.* 28 350–356.

[B16] Dunphy C. M. Gouhier T. C. Chu N. D. Vollmer S. V. (2019). Structure and stability of the coral microbiome in space and time. *Sci. Rep.* 9:6785. 10.1038/s41598-019-43268-6 31043671 PMC6494856

[B17] Fabina N. S. Putnam H. M. Franklin E. C. Stat M. Gates R. D. (2013). Symbiotic specificity, association patterns, and function determine community responses to global changes: defining critical research areas for coral-symbiodinium symbioses. *Glob. Chan. Biol.* 19 3306–3316. 10.1111/gcb.12320 23847174

[B18] Fitt W. K. McFarland F. K. Warner M. E. Chilcoat G. C. (2000). Seasonal patterns of tissue biomass and densities of symbiotic dinoflagellates in reef corals and relation to coral bleaching. *Limnol. Oceanograp.* 45 677–685. 10.4319/lo.2000.45.3.0677

[B19] Gajigan A. P. Diaz L. A. Conaco C. (2017). Resilience of the prokaryotic microbial community of acropora digitifera to elevated temperature. *Microbiologyopen* 6:e478. 10.1002/mbo3.478 28425179 PMC5552946

[B20] Garren M. Raymundo L. Guest J. Harvell C. Azam F. (2009). Resilience of coral-associated bacterial communities exposed to fish farm effluent. *Plos One* 4:e7319. 10.1371/journal.pone.0007319 19806190 PMC2751826

[B21] Glynn P. W. (1993). Coral reef bleaching: ecological perspectives. *Coral Reefs* 12 1–17. 10.1007/BF00303779

[B22] Grottoli A. G. Dalcin Martins P. Wilkins M. J. Johnston M. D. Warner M. E. Cai W. (2018). Coral physiology and microbiome dynamics under combined warming and ocean acidification. *PLoS One* 13:e191156. 10.1371/journal.pone.0191156 29338021 PMC5770069

[B23] Grottoli A. G. Rodrigues L. J. Juarez C. (2004). Lipids and stable carbon isotopes in two species of hawaiian corals, porites compressa and montipora verrucosa, following a bleaching event. *Mar. Biol.* 145 621–631. 10.1007/s00227-004-1337-3

[B24] Guo X. Feng J. Shi Z. Zhou X. Yuan M. Tao X. (2018). Climate warming leads to divergent succession of grassland microbial communities. *Nat. Clim. Chan.* 8 813–818. 10.1038/s41558-018-0254-2

[B25] Hernandez-Agreda A. Gates R. D. Ainsworth T. D. (2017). Defining the core microbiome in corals’ microbial soup. *Trends Microbiol.* 25 125–140. 10.1016/j.tim.2016.11.003 27919551

[B26] Horner-Devine M. C. Bohannan B. J. M. (2006). Phylogenetic clustering and overdispersion in bacterial communities. *Ecology* 87 S100–S108. 10.1890/0012-9658200687 16922306

[B27] Hughes T. P. Kerry J. T. Álvarez-Noriega M. Álvarez-Romero J. G. Anderson K. D. Baird A. H. (2017). Global warming and recurrent mass bleaching of corals. *Nature* 543 373–377. 10.1038/nature21707 28300113

[B28] Hughes T. P. Kerry J. T. Baird A. H. Connolly S. R. Dietzel A. Eakin C. M. (2018). Global warming transforms coral reef assemblages. *Nature* 556:492. 10.1038/s41586-018-0041-2 29670282

[B29] Hume B. Angelo C. Burt J. Baker A. C. Riegl B. Wiedenmann J. (2013). Corals from the persian/arabian gulf as models for thermotolerant reef-builders: prevalence of clade c3 symbiodinium, host fluorescence and ex situ temperature tolerance. *Mar. Pollut. Bull.* 72 313–322. 10.1016/j.marpolbul.2012.11.032 23352079

[B30] Jiang L. Sun Y. F. Zhang Y. Y. Zhou G. W. Huang H. (2017). Impact of diurnal temperature fluctuations on larval settlement and growth of the reef coral pocillopora damicornis. *Biogeosciences* 14 5741–5752.

[B31] Jiao S. Chen W. Wei G. (2021). Linking phylogenetic niche conservatism to soil archaeal biogeography, community assembly and species coexistence. *Glob. Ecol. Biogeograp.* 30 1488–1501. 10.1111/geb.13313

[B32] Jiao S. Yang Y. Xu Y. Zhang J. Lu Y. (2020). Balance between community assembly processes mediates species coexistence in agricultural soil microbiomes across eastern china. *ISME J.* 14 202–216. 10.1038/s41396-019-0522-9 31611655 PMC6908645

[B33] Jonathan M. Chase Jonathan A. Myers. (2011). Disentangling the importance of ecological niches from stochastic processes across scales. *Philos. Trans. Royal Soc. B: Biol. Sci.* 366 2351–2363. 10.1098/rstb.2011.0063 21768151 PMC3130433

[B34] Jung E. M. U. Stat M. Thomas L. Koziol A. Schoepf V. (2021). Coral host physiology and symbiont dynamics associated with differential recovery from mass bleaching in an extreme, macro-tidal reef environment in northwest australia. *Coral Reefs* 40 893–905. 10.1007/s00338-021-02094-x

[B35] Kelly L. W. Williams G. J. Barott K. L. Carlson C. A. Dinsdale E. A. Edwards R. A. (2014). Local genomic adaptation of coral reef-associated microbiomes to gradients of natural variability and anthropogenic stressors. *Proc. Nat. Acad. Sci.* 111:10227. 10.1073/pnas.1403319111 24982156 PMC4104888

[B36] Levas S. J. Grottoli A. G. Hughes A. Osburn C. L. Matsui Y. (2013). Physiological and biogeochemical traits of bleaching and recovery in the mounding species of coral porites lobata: implications for resilience in mounding corals. *PLoS One* 8:e63267. 10.1371/journal.pone.0063267 23658817 PMC3642184

[B37] Mao-Jones J. Ritchie K. B. Jones L. E. Ellner S. P. (2010). How microbial community composition regulates coral disease development. *PLoS Biol.* 8:e1000345. 10.1371/journal.pbio.1000345 20361023 PMC2846858

[B38] McCauley M. Jackson C. R. Goulet T. L. (2020). Microbiomes of caribbean octocorals vary over time but are resistant to environmental change. *Front. Microbiol.* 11:1272. 10.3389/fmicb.2020.01272 32595627 PMC7304229

[B39] Meron D. Atias E. Iasur Kruh L. Elifantz H. Minz D. Fine M. (2011). The impact of reduced ph on the microbial community of the coral acropora eurystoma. *ISME J.* 5 51–60. 10.1038/ismej.2010.102 20668489 PMC3105665

[B40] Mo Y. Peng F. Gao X. Xiao P. Logares R. Jeppesen E. (2021). Low shifts in salinity determined assembly processes and network stability of microeukaryotic plankton communities in a subtropical urban reservoir. *Microbiome* 9:128. 10.1186/s40168-021-01079-w 34082826 PMC8176698

[B41] Mueller J. S. Schupp P. J. (2020). Shading by marine litter impairs the health of the two indo-pacific scleractinian corals porites rus and pavona cactus. *Mar. Pollut. Bull.* 158:111429. 10.1016/j.marpolbul.2020.111429 32753213

[B42] Nemergut D. R. Schmidt S. K. Fukami T. ONeill S. P. Bilinski T. M. Stanish L. F. (2013). Patterns and processes of microbial community assembly. *Microbiol. Mol. Biol. Rev.* 77 342–356. 24006468 10.1128/MMBR.00051-12 PMC3811611

[B43] Pearman J. K. Aylagas E. Voolstra C. R. Anlauf H. Villalobos R. Carvalho S. (2019). Disentangling the complex microbial community of coral reefs using standardized autonomous reef monitoring structures (arms). *Mol. Ecol.* 28 3496–3507. 10.1111/mec.15167 31281998 PMC6851789

[B44] Peixoto R. S. Rosado P. M. Assis L. Rosado A. S. Bourne D. G. (2017). Beneficial microorganisms for corals (bmc): proposed mechanisms for coral health and resilience. *Front. Microbiol.* 8:341. 10.3389/fmicb.2017.00341 28326066 PMC5339234

[B45] Pogoreutz C. Rädecker N. Cárdenas A. Gärdes A. Wild C. Voolstra C. R. (2018). Dominance of endozoicomonas bacteria throughout coral bleaching and mortality suggests structural inflexibility of the pocillopora verrucosa microbiome. *Ecol. Evol.* 8 2240–2252. 10.1002/ece3.3830 29468040 PMC5817147

[B46] Pontarp M. Canbäck B. Tunlid A. Lundberg P. (2012). Phylogenetic analysis suggests that habitat filtering is structuring marine bacterial communities across the globe. *Microbial. Ecol.* 64 8–17. 10.1007/s00248-011-0005-7 22286378 PMC3375428

[B47] Pootakham W. Mhuantong W. Putchim L. Yoocha T. Sonthirod C. Kongkachana W. (2018). Dynamics of coral-associated microbiomes during a thermal bleaching event. *Microbiologyopen* 7:e604. 10.1002/mbo3.604 29573244 PMC6182559

[B48] Price J. T. McLachlan R. H. Jury C. P. Toonen R. J. Wilkins M. J. Grottoli A. G. (2021). Effect of species, provenance, and coral physiology on the composition of hawaiian coral-associated microbial communities. *Coral Reefs* 40 1537–1548. 10.1007/s00338-021-02164-0

[B49] Qlca B. Hwha B. Zzy B. Cyl B. Batn B. Aqs A. (2020). Deterministic selection dominates microbial community assembly in termite mounds. *Soil Biol. Biochem.* 152:73.

[B50] Roder C. Arif C. Bayer T. Aranda M. Daniels C. Shibl A. (2014). Bacterial profiling of white plague disease in a comparative coral species framework. *ISME J.* 8 31–39. 10.1038/ismej.2013.127 23924783 PMC3869008

[B51] Romdhane S. Spor A. Aubert J. Bru D. Breuil M. Hallin S. (2021). Unraveling negative biotic interactions determining soil microbial community assembly and functioning. *ISME J.* 2021:769. 10.1038/s41396-021-01076-9 34321619 PMC8692615

[B52] Rosset S. Wiedenmann J. Reed A. J. D’Angelo C. (2017). Phosphate deficiency promotes coral bleaching and is reflected by the ultrastructure of symbiotic dinoflagellates. *Mar. Pollut. Bull.* 118 180–187. 10.1016/j.marpolbul.2017.02.044 28242282 PMC5441187

[B53] Shi T. Niu G. Kvitt H. Zheng X. Qin Q. Sun D. (2021). Untangling its2 genotypes of algal symbionts in zooxanthellate corals. *Mol. Ecol. Res.* 21 137–152. 10.1111/1755-0998.13250 32876380

[B54] Shinzato C. Shoguchi E. Kawashima T. Hamada M. Hisata K. Tanaka M. (2011). Using the acropora digitifera genome to understand coral responses to environmental change. *Nature* 476 320–323. 10.1038/nature10249 21785439

[B55] Sloan W. T. Lunn M. Woodcock S. Head I. M. Nee S. Curtis T. P. (2006). Quantifying the roles of immigration and chance in shaping prokaryote community structure. *Environ. Microbiol.* 8 732–740. 10.1111/j.1462-2920.2005.00956.x 16584484

[B56] Davies P. S. (1989). Short-term growth measurements of corals using an accurate buoyant weighing technique. *Mar. Biol.* 101 389–395. 10.1007/BF00428135

[B57] Stegen J. C. Lin X. Fredrickson J. K. Chen X. Kennedy D. W. Murray C. J. (2013). Quantifying community assembly processes and identifying features that impose them. *ISME J.* 7 2069–2079. 10.1038/ismej.2013.93 23739053 PMC3806266

[B58] Stegen J. C. Lin X. Konopka A. E. Fredrickson J. K. (2012). Stochastic and deterministic assembly processes in subsurface microbial communities. *ISME J.* 6 1653–1664. 10.1038/ismej.2012.22 22456445 PMC3498916

[B59] Thomas L. López E. H. Morikawa M. K. Palumbi S. R. (2019). Transcriptomic resilience, symbiont shuffling, and vulnerability to recurrent bleaching in reef-building corals. *Mol. Ecol.* 28 3371–3382. 10.1111/mec.15143 31177587

[B60] Thompson J. R. Rivera H. E. Closek C. J. MNica M. (2014). Microbes in the coral holobiont: partners through evolution, development, and ecological interactions. *Front. Cell. Infect. Microbiol.*:4:176. 10.3389/fcimb.2014.00176 25621279 PMC4286716

[B61] Tripathi B. M. Stegen J. C. Kim M. Dong K. Adams J. M. Lee Y. K. (2018). Soil ph mediates the balance between stochastic and deterministic assembly of bacteria. *ISME J.* 12:82. 10.1038/s41396-018-0082-4 29515169 PMC5864241

[B62] van de Water J. A. J. M. Allemand D. Ferrier-Pagès C. (2018). Host-microbe interactions in octocoral holobionts - recent advances and perspectives. *Microbiome* 6:64. 10.1186/s40168-018-0431-6 29609655 PMC5880021

[B63] van Oppen M. J. H. Blackall L. L. (2019). Coral microbiome dynamics, functions and design in a changing world. *Nat. Rev. Microbiol.* 17 557–567. 10.1038/s41579-019-0223-4 31263246

[B64] Wall C. B. Ritson-Williams R. Popp B. N. Gates R. D. (2019). Spatial variation in the biochemical and isotopic composition of corals during bleaching and recovery. *Limnol. Oceanograp.* 64 2011–2028. 10.1002/lno.11166 31598010 PMC6774332

[B65] Wang J. Shen J. Wu Y. Tu C. Soininen J. Stegen J. C. (2013). Phylogenetic beta diversity in bacterial assemblages across ecosystems: deterministic versus stochastic processes. *ISME J.* 7 1310–1321. 10.1038/ismej.2013.30 23446837 PMC3695296

[B66] Wang Q. Garrity G. M. Tiedje J. M. Cole J. R. (2007). Nave bayesian classifier for rapid assignment of rrna sequences into the new bacterial taxonomy. *Appl. Environ. Microbiol.* 73:5261. 10.1128/AEM.00062-07 17586664 PMC1950982

[B67] Wijgerde T. van Ballegooijen M. Nijland R. van der Loos L. Kwadijk C. Osinga R. (2020). Adding insult to injury: effects of chronic oxybenzone exposure and elevated temperature on two reef-building corals. *Sci. Total Environ.* 733:139030. 10.1016/j.scitotenv.2020.139030 32446051

[B68] Wooldridge Scott A. (2014). Differential thermal bleaching susceptibilities amongst coral taxa: re-posing the role of the host. *Coral Reefs* 33 15–27.

[B69] Xiao R. Zhou H. Chen C. M. Cheng H. Li H. Xie J. (2018). Transcriptional responses of acropora hyacinthus embryo under the benzo(a)pyrene stress by deep sequencing. *Chemosphere* 206:387. 10.1016/j.chemosphere.2018.04.149 29754063

[B70] Xu L. Yu K. Li S. Liu G. Tao S. Shi Q. (2017). Interseasonal and interspecies diversities of symbiodinium density and effective photochemical efficiency in five dominant reef coral species from luhuitou fringing reef, northern south china sea. *Coral Reefs* 36 477–487. 10.1007/s00338-016-1532-y

[B71] Yu X. Yu K. Huang W. Liang J. Qin Z. Chen B. (2020). Thermal acclimation increases heat tolerance of the scleractinian coral acropora pruinosa. *Sci. Total Environ.* 733:139319. 10.1016/j.scitotenv.2020.139319 32446076

[B72] Yuan M. M. Guo X. Wu L. Zhang Y. Xiao N. Ning D. (2021). Climate warming enhances microbial network complexity and stability. *Nat. Clim. Chan.* 11 343–348. 10.1038/s41558-021-00989-9

[B73] Yun Y. Gui Z. Xie J. Chen Y. Tian X. Li G. (2021). Stochastic assembly process dominates bacterial succession during a long-term microbial enhanced oil recovery. *Sci. Total Environ.* 790:148203. 10.1016/j.scitotenv.2021.148203 34380257

[B74] Zaneveld J. R. Burkepile D. E. Shantz A. A. Pritchard C. E. McMinds R. Payet J. P. (2016). Overfishing and nutrient pollution interact with temperature to disrupt coral reefs down to microbial scales. *Nat. Commun.* 7:11833. 10.1038/ncomms11833 27270557 PMC4899628

[B75] Zaneveld J. R. McMinds R. Vega Thurber R. (2017). Stress and stability: applying the anna karenina principle to animal microbiomes. *Nat. Microbiol.* 2:17121. 10.1038/nmicrobiol.2017.121 28836573

[B76] Zhang Y. Yang Q. Zhang Y. Ahmad M. Ling J. Tang X. (2021). Shifts in abundance and network complexity of coral bacteria in response to elevated ammonium stress. *Sci. Total Environ.* 768:144631. 10.1016/j.scitotenv.2020.144631 33434804

[B77] Zhenjun Q. Kefu B. Chen Y. (2019). Diversity of symbiodiniaceae in 15 coral species from the southern south china sea: potential relationship with coral thermal adaptability. *Front. Microbiol.* 10:2343. 10.3389/fmicb.2019.02343 31681208 PMC6813740

[B78] Zhou J. Z. Ning D. L. (2017). Stochastic community assembly: does it matter in microbial ecology? *Microbiol. Mol. Biol. Rev.* 81:17. 10.1128/MMBR.00002-17 29021219 PMC5706748

[B79] Zhu J. Hong Y. Zada S. Hu Z. Wang H. (2018). Spatial variability and co-acclimation of phytoplankton and bacterioplankton communities in the pearl river estuary, china. *Front. Microbiol.* 9:2503. 10.3389/fmicb.2018.02503 30405565 PMC6206238

[B80] Zhu W. T. Xia J. Q. Ren Y. X. Xie M. Yin H. Liu X. (2021). Coastal corals during heat stress and eutrophication: a case study in northwest hainan coastal areas. *Mar. Pollut. Bull.* 173:113048. 10.1016/j.marpolbul.2021.113048 34678546

[B81] Ziegler M. Eguíluz V. M. Duarte C. M. Voolstra C. R. (2018). Rare symbionts may contribute to the resilience of coral–algal assemblages. *ISME J.* 12 161–172. 10.1038/ismej.2017.151 29192903 PMC5739009

[B82] Ziegler M. Seneca F. O. Yum L. K. Palumbi S. R. Voolstra C. R. (2017). Bacterial community dynamics are linked to patterns of coral heat tolerance. *Nat. Commun.* 8:14213. 10.1038/ncomms14213 28186132 PMC5309854

